# Design and validation of equations for weight estimation in adolescents

**DOI:** 10.1371/journal.pone.0273824

**Published:** 2023-02-02

**Authors:** Daniel Meyer Coracini, Cláudia Rucco Penteado Detregiachi, Sandra Maria Barbalho, Daniel De Bortoli Teixeira

**Affiliations:** 1 Postgraduate Program in Structural and Functional Interactions in Rehabilitation - University of Marília (UNIMAR), Marília, São Paulo, Brazil; 2 Department of Nutrition, University of Marília (UNIMAR), Marília, São Paulo, Brazil; 3 Department of Biochemistry and Pharmacology, School of Medicine, University of Marília (UNIMAR), Marília, São Paulo, Brazil; 4 School of Food and Technology of Marilia (FATEC), Marilia, São Paulo, Brazil; Hospital Privado Universitario de Córdoba: Hospital Privado Centro Medico de Cordoba, ARGENTINA

## Abstract

**Introduction:**

Measuring weight is difficult to be carried out in bedridden people, with physical deformity or in emergency units. Under these circumstances, one option is to estimate the weight.

**Objectives:**

The aim of this study is to propose and validate equations for estimating the weight of Brazilian adolescents based on anthropometric variables related to body weight.

**Methods:**

The study was developed based on a database created from data collection of a primary project, which had information from 662 Brazilian adolescents (10 to 19 years old). Based on the variables sex, age (days), weight (kg), height (m) and neck circumference (NC) (cm), equations for estimating weight of adolescents were proposed. The formulas were proposed after performing multiple linear regression models and subsequently tested and validated using appropriate statistical tests, considering 99% confidence.

**Results:**

Two formulas were generated, the “Rucco Formulas—Adolescents”, one for girls: -131.63091 + (0.00209 × A) + (37.57813 × H) + (3.71482 x NC) and another for boys: - 15.2854 + (-0.00414 × A)+ (14.30315 × H^2^)+ (0.04888 x NC^2^). Statistical test (R^2^) indicated that the proposed formulas are suitable for estimating weight. Low values of REQM and high values of CCI (> 0.8) also reinforce the quality of the proposed formulas.

**Conclusions:**

The current weight of adolescents can be estimated with adequate accuracy and precision using sex-specific “Rucco Formulas—Adolescents”, generated from regression models using only three predictor variables.

## Introduction

Anthropometric measurements represent one of the most used nutritional assessment methods because it is non-invasive, low-cost, practical and easy to apply [1]. The most used anthropometric measures in the pediatric age group are weight, height, head circumference, and abdominal circumference [2].

Furthermore, bodyweight is also an important data in the clinical area, being used to accurately calculate dosages of drugs and fluids, as well as in the correct selection of medical equipment [3] and ventilation settings [4, 5]. However, weight measurements are difficult to perform in bedridden people. Another aspect to consider is that in emergency units, where time is a critical determinant, there is usually no possibility of assessing weight due to logistical characteristics and practical reasons of the location [3].

An alternative in these circumstances is the weight estimate, for which there are different methodologies. One of them is the method of counting the fingers of both hands, which can also be represented by an age-based equation [6]. Age-based formulas are the oldest methods for estimating weight, with several equations available [7–14].

There are formulas based on body measurements to estimate the actual weight of children and adolescents such as mid-arm circumference (MAC), humerus length, and body height [4, 5, 15–21].

We can assume that the weight estimation principles used in both age-based equations and the finger counting methodare more associated with the expected weight than the actual weight, since they do not consider body measures with the explanatory power of body weight. However, in some aspects, the actual weight is the most critical evaluation parameter, since it is necessary to medication dosages or in the correct selection of medical equipment. Moreover, it is also necessary to identifyweight disorders as early as possible.

Other body measurements can also be associated with actual body weight, and the study of new equations can bring a new scenario for weight estimation both in the clinical area and in primary care. Thus, this study aimed to propose and validate equations for estimating the weight of Brazilian adolescents based on anthropometric variables related to body weight, including the neck circumference measure, never before used in weight estimation formulas, given its practicality and ease of collection.

## Materials and methods

This was a primary, observational, cross-sectional, quantitative, and analytical study developed based on a database created in 2018 related to a preliminary project approved by the Research Ethics Committee of the University of Marília. This database contains data from 662 adolescents aged 10 to 19 years, on which it was possible to apply statistical tests. For the construction of the database, the minimum number of samples was determined [22] considering a descriptive study of the variable weight. The parameters used for the calculation were: 99% (confidence level); 5 kg (total width of confidence interval); and, 20 kg (standard deviation of the variable), totaling a minimum sample size of 425 students.

Tais adolescents foram recrutados em public and private schools in the city of Marília/SP/Brazil, with prior authorization from the school director. In these educational institutions, all adolescent students were invited to participate by sending an invitation letter to their parents or guardians. Those who agreed with the adolescent’s participation returned the signed Free and Informed Consent Form, plus information about medications in use and the presence of disease. Adolescents who had the consent of their parents or guardians but who had diabetes, thyroid disease or anomalies in the head and neck region, or who were still in the gestational period were excluded.

From the database, information on sex, birth date, height (m) and neck circumference (NC) (cm) were used in the tests of the explanatory power of variations in the measured weight. This is justified based on proposing formulas for estimating weight with the least number of variables to meet the need for speed and practicality in clinical and emergency units. In addition, the measured weight (kg) was also used as a reference. The measurement of weight and height was performed according to the recommended techniques [23, 24]. Body weight was collected using a platform-type digital scale, with a maximum capacity of 200 kg. Height was measured with the aid of a portable collapsible statemeter. The NC was obtained with the adolescent standing and the head elevated to acquire the midpoint between the lower part of the chin and the manubrium sternal bone. Afterwards, the adolescent’s head was placed in the Frankfurt position and an inextensible tape measure was applied perpendicularly around the neck, exactly on top of the previously obtained midpoint. When the male adolescent had an evident laryngeal prominence, the measurement was taken just below it. This technique was adapted from the one used by Preis et al. [25] and Vasques et al. [26]. The research team responsible for collecting the anthropometric data was trained to determine the agreement of the measurements taken by its components in relation to an experienced professional. To evaluate the calibration of the team, the statistical methods of precision and accuracy proposed by Habicht, 1974 [27] were used.

Age was calculated in days by the difference between the date of assessment of the measures and the birth date. Information on the sexual maturity stage that was available in the database was also included.

The nutritional diagnosis of adolescents was determined using the anthropometric indicator of body mass index (BMI) for age, which was classified based on the reference standards of the Ministry of Health [1]. In the preliminary study, the stage of sexual maturity was defined by self-assessment, according to the criteria defined by [28]. Although it is a subjective evaluation and with some limitations, it is the most suitable for population surveys due to the difficulty of being obtained by medical evaluation [29]. Stage one corresponds to prepubertal development and growth, while stages two to four correspond to the progression from puberty to full maturity (stage five) [28].

The database was separated by sex and divided into two parts, with 70% data reserved for the proposal of formulas (n = 462) and 30% (n = 200) for the validation step. This separation of the database was performed using the randomization method available in the spreadsheet. Additionally, the validation was also performed considering the stages of Tanner’s sexual maturity.

Prior to constructing the formulas, variables were described through the estimation of the means, median, standard deviation, minimum and maximum values of each population (girls and boys) evaluated. Subsequently, Student’s *t*-test or Mann Whitney test was applied to check for differences between the values of the variables in these populations. The choice of tests was based on the presence/absence of normality indicated by the Kolmogorov-Smirnov test. The nutritional diagnosis was compared between the sexes, using the Chi-square test.

Equations for weight estimation were obtained using multiple stepwise linear regression considering the level of significance of 1% for the input and output of selected variables (age, height, and NC). To determine the equations, we sought to evaluate possible linear and quadratic relationships between the weight and the input variables.

The quality of the estimative of weight were performed with descriptive analysis, mean difference between the measured and estimated values and the respective 99% confidence interval (99% CI). In the concordance analysis, the Bland-Altman plot, the coefficient of determination (R^2^), root mean square error (RMSE), intraclass correlation coefficient (ICC), and its respective 99% CI were used. To verify the magnitude of errors in each equation, the graphical analysis of standardized residuals and the Shapiro-Wilk test were used to check the adequacy of the proposed model with the necessary statistical assumptions [30]. The R^2^ indicates the percentage of the variation in the measured weight that the proposed estimation model explains. According to Lima et al. [30], values greater than 0.7 indicate that the equation can be considered adequate for estimating weight. For all analyses, 1% was adopted as the significance value. The relative frequency of errors ((predicted–observed)/observed) was also verified as a function of age and sex, with an error below 10% being considered acceptable.

After defining the formulas, they were tested and validated using the rest of the data (30%). For validation were used the mean difference between the measured and estimated values and the respective 99% confidence interval (99% CI), the coefficient of determination (R^2^), root mean square error (RMSE), intraclass correlation coefficient (ICC), and its respective 99% CI were used. The software R [31] was used for statistical analyses.

This research project was approved by the Research Ethics Committee of the University of Marília (protocol number 3,682,722).

## Results

### Proposed weight estimation formulas

Data from 462 adolescents (70% of total sample) were used, of which 64% were girls. The variables Age and BMI presented normal distribution (p>0.01), while the variables weight, height and NC did not present normal distribution (p<0.01). The two groups, girls and boys, did not differ in BMI (Table 1). However, the boys had higher weight, height, and NC measurement (p <0.01).

**Table 1:** Description of the sample of adolescents used in the proposal of formulas for weight estimation.

Variables		Girls (n = 296)	Boys (n = 166)	p-value
		Mean±standard deviation(median)		
**Age (years)**		14.5±2.1 (15.0)	14.8±2.2 (15.0)	0.2241*
**Weight (kg)**		57.0±13.7 (55.2)	62.4±16.3 (59.1)	0.0002**
**Height (m)**		1.6±0.1 (1.6)	1.7±0.1 (1.7)	<0.0001**
**Neck circumference (cm)**		31.4±2.5 (31.0)	34.5±3.2 (34.4)	<0.0001**
**BMI (kg/m^2^)**		22.3±4.8 (21.5)	22.0±4.2 (20.9)	0.5852*
Nutritional diagnosis**** **n (%)**	**Lean**	7 (2%)	7 (4%)	0.323***
	**Normal weight**	186 (63%)	98 (59%)	
	**Overweight**	62 (21%)	30 (18%)	
	**Obesity**	41 (14%)	31 (19%)	

Kg = kilos. m = meters. cm = centimeters.

*Student’s *t*-test of independence.

**Mann Whitney: independent samples.

***Chi-square test.

****Classification according to Brazil [1].

The determination of the nutritional diagnosis indicated a predominance of eutrophy; although 35% of girls and 37% of boys were overweight (overweight or obese), a diagnosis also present in 36% general sample (Table 1).

The differences observed between girls and boys for some anthropometric measures (Table 1) support different formulas for estimating weight for both sexes.

Multiple linear regression models were applied to the variables age, height and NC to develop formulas for estimating weight. Based on the value of R^2^, for the girls’ group, these variables together presented explanatory power of the measured weight variation greater than 70% (0.7), while for the boys’ group, the combined explanatory power was greater than 75% (0.75) (Table 2).

**Table 2:** Multiple linear regression models for estimating weight (kg) in adolescents.

Variables	Estimated parameter	SE	p-value	R^2^
**Girls (n = 296)**				
**Intercept**	-131.63091	7.75506	<0.0001	
**NC (cm)**	3.71482	0.17865	<0.0001	0.6474
**Height (m)**	37.57813	5.28514	<0.0001	0.7309
**Age (days)**	0.00209	0.00049182	<0.0001	0.7476
**Boys (n = 166)**				
**Intercept**	-15.28545	4.10176	0.0003	
**NC**^**2**^ **(cm)**	0.04888	0.00344	<0.0001	0.7109
**Height**^**2**^ **(m)**	14.30315	2.26142	<0.0001	0.7437
**Age (days)**	-0.00414	0.00095778	<0.0001	0.7718

NC = neck circumference. m = meters. cm = centimeters. SE = standard error; R^2^ = coefficient of determination.

The variable NC was the first selected by the model in both cases, being responsible for 64.74% (R^2^ = 0.6474) of the variation in the weight of the group of girls and 71.09% (R^2^ = 0.7109) in the group of boys, the latter using the squared NC values. Height was the second variable to enter the model, being responsible for an additional explanation of 8.35% and 3.28% for girls and boys (squared height), respectively. Finally, the age variable contributes with an additional explanation of 1.67% (girls) and 2.81% (boys) (Table 2).

These data support the possibility of proposing formulas for estimating weight in adolescents with a small number of variables, in addition to being easily obtainable in clinical or emergency practice.

**Fig 1:**
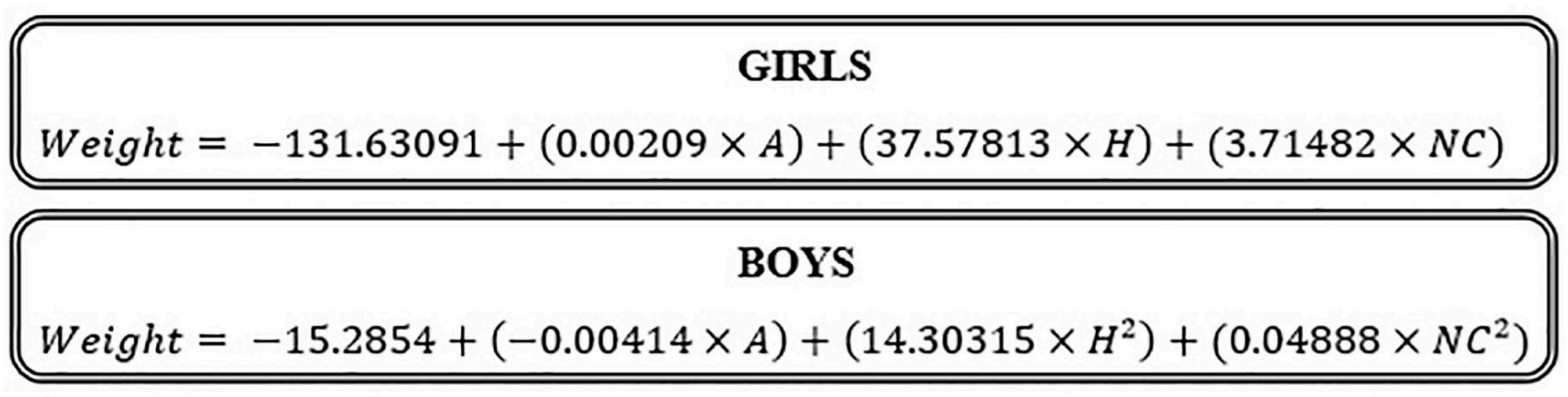
Proposed formulas for estimating adolescent weight, according to sex. Weight (kg). A = age (days). H = height (m). NC = neck circumference (cm).

The graphic summary in Fig 1 shows the formulas developed to estimate weight in adolescents, as a function of sex, according to data in (Table 2). These were called “Rucco Formulas—Adolescents”.

The final model for both groups reached R^2^ values above 0.70, 0.75 for girls and 0.77 for boys. It is worth mentioning that R^2^ values greater than 0.7 indicate that the proposed formula can be considered adequate for estimating weight [30].

**Fig 2:**
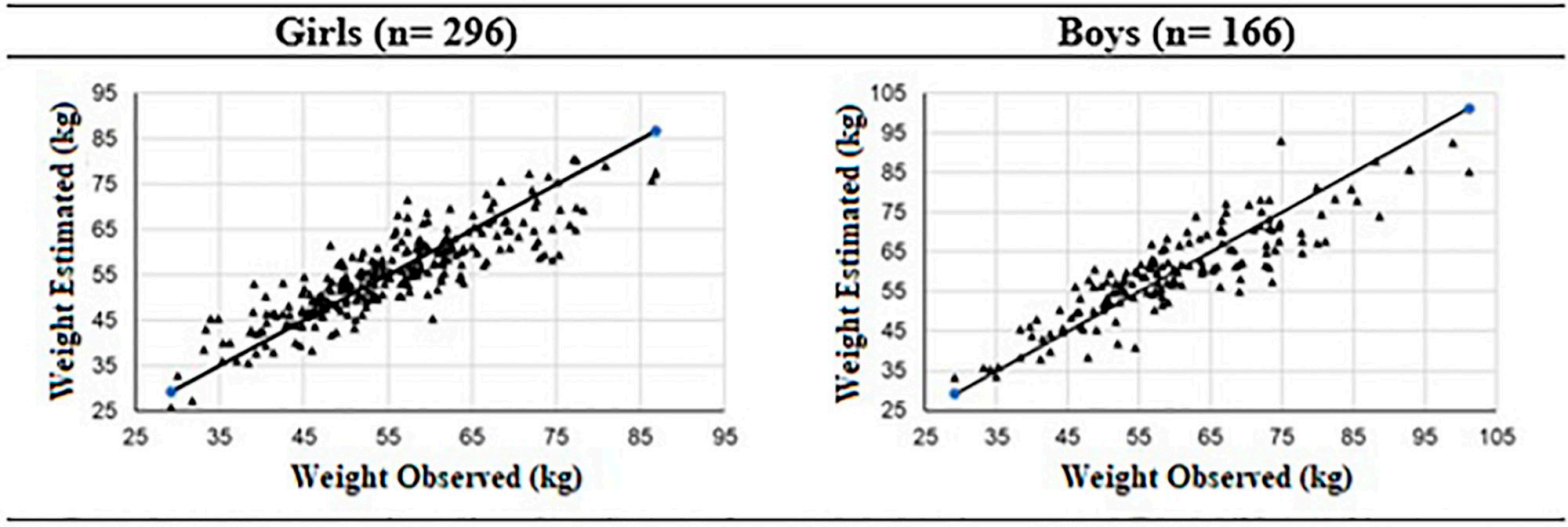
Weight values observed and estimated by the proposed formulas. kg = kilos.

Through the data used to construct the formulas, a preliminary check of the quality of the estimates of the proposed models was performed Table 3 and Fig 2. For both groups, the mean values of the measured and estimated weights are practically coincident, presenting only a greater homogeneity in estimated data (lower values of standard deviation) concerning the measured data, demonstrating a slight smoothing of the estimates. The low values of RMSE and the high values of CCI (> 0.8) also reinforce the quality of the proposed formulas.

**Table 3:** Quality of the estimates of the proposed formulas for estimating adolescent weight.

	Girls (n = 296)	Boys (n = 166)
**Mean (SD) of the measured weight (kg)**	55.2 (10.8)	60.3 (13.3)
**Mean (SD) of the estimated weight (kg)**	55.2 (9.33)	60.3 (11.7)
**Difference (SD) between mean values of the measured and estimated weights (kg)**	7.3×10^−7^ (5.4)	1.3 × 10^−6^ (6.4)
**99% CI difference**	(-0.85; 0.85)	(-1.33; 1.33)
**R** ^**2**^	0.75	0.77
**RMSE**	5.41	6.3
**ICC (p-value)**	0.86 (4.81×10^−81^)	0.87 (1.7×10^−50^)
**99% CI (ICC)**	(0.81; 0.89)	(0.81; 0.91)

SD = standard deviation. 99% CI = 99% difference confidence interval. R^2^ = coefficient of determination. RMSE = root mean square error. ICC = intraclass correlation coefficient.

**Fig 3:**
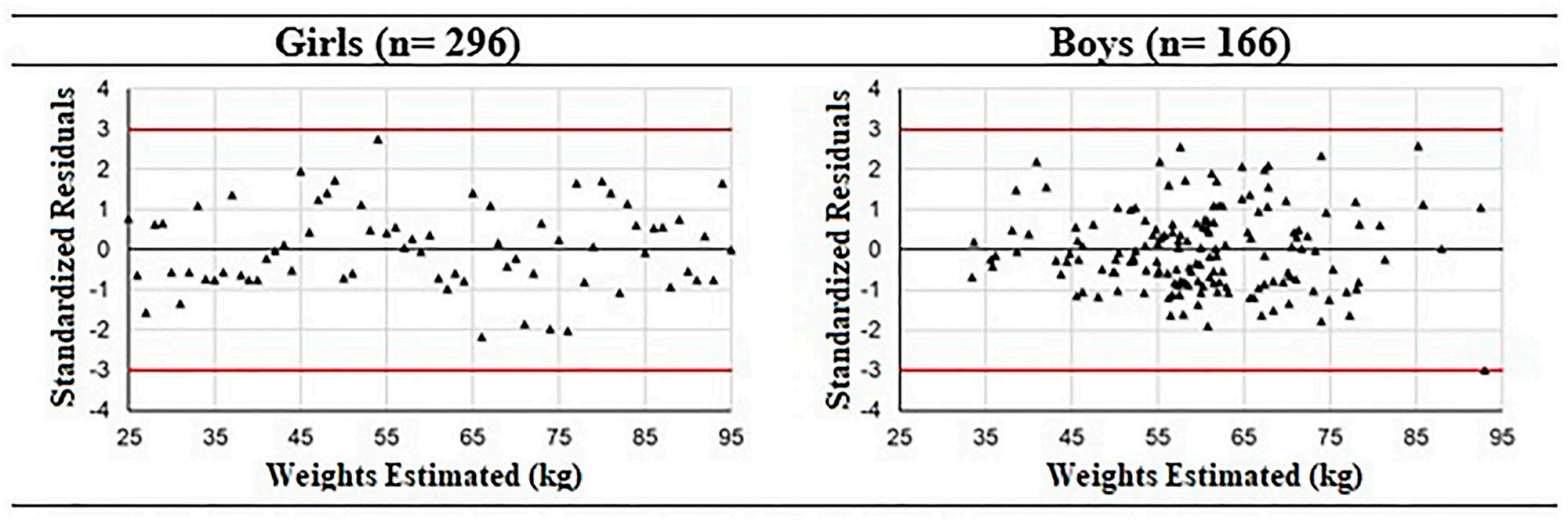
Graphical analysis of formula residuals for adolescent weight estimates. kg = kilos.

The assumptions of the regression analyses were tested to observe the validity of the proposed formulas. The Shapiro-Wilk test confirmed the residual normality of the models proposed for girls (p = 0.0930) and boys (p = 0.0374). The graphical analysis of the standardized residuals as a function of the weights estimated by the proposed equations (Fig 3) shows the presence of homogeneity in residual variances, as well as the absence of extreme values and outliers, thus meeting the assumptions of the regression analyses and, consequently, the validity of formulas.

**Fig 4:**
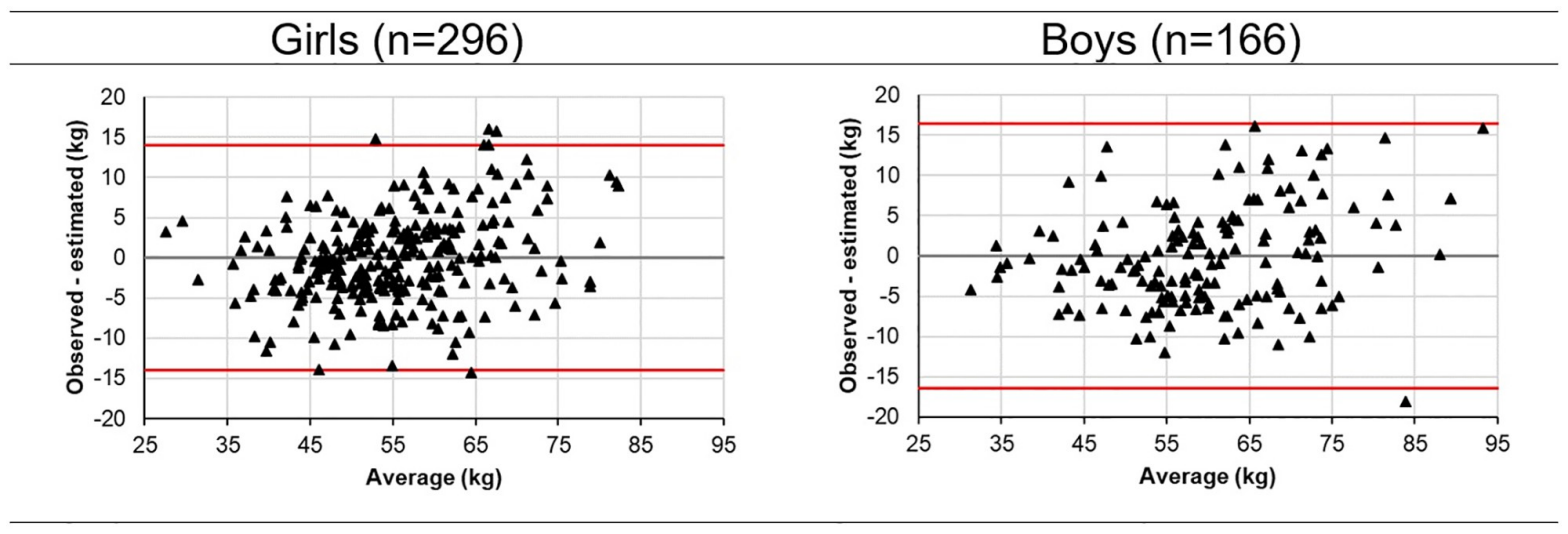
Bland-Altman plots for weight and estimate weight. Label: Bland-Altman plot for the agreement between estimated weight and observed weight. Data triangles represent the individual children. The mean is represented by the centered line, and the upper and lower lines represent the standard deviation and the 99% CIs for the agreement between the 2 methods.

The Bland-Altman plots showed a strong consistency between the observed and the predicted. Between the weight observed and the estimated weight (Fig 4) the limits of agreement (Reference Range for difference), for the girls was -13.989 to 13.989 and for the boys was -16,414 to 16,414. The Mean difference was -0.000 (CI -0.85 to 0.85) and -0.000 (CI -1.33 to 1.33), for girls and boys, respectively. It is also noted that the percentage of correct answers (errors below 10%) shows little variation according to age and sex (Table 4), and, in general, 84% of the estimated samples are located within the tolerable limit (10%) for both sexes.

**Table 4:** Frequency distribution of estimation errors below 10% and the relationship with age and sex.

Age (year)	Girls	Boys
**10**	1 (100.0%)	3 (100.0%)
**11**	12 (75.0%)	6 (85.7%)
**12**	31 (83.8%)	18 (81.8%)
**13**	25 (83.3%)	12 (80.0%)
**14**	21 (95.5%)	8 (88.9%)
**15**	45 (86.5%)	20 (76.9%)
**16**	48 (80.0%)	18 (90.0%)
**17**	32 (86.5%)	34 (85.0%)
**18**	15 (88.2%)	11 (91.7%)
**19**	2 (66.7%)	1 (50.0%)
**Total**	232 (84.4%)	131 (84.0%)

### Validation of the proposed formulas for weight estimation

For the formula validation stage, data from 200 adolescents (30% of the sample) from the original database were used.

The comparison between the data from the sample of adolescents used for the proposal of the formulas with that used in the validation indicates the absence of a significant difference for the variables analyzed (Table 5), confirming that the process of random selection of samples for the proposal and samples for validation was performed correctly.

**Table 5:** Description of the two samples of adolescents used in the proposal and validation of the formulas developed for weight estimation.

Variables		Sample used in the proposal (n = 462)	Sample used in the validation (n = 200)	p-value
		Mean ± standard deviation(mediana)		
**Age (years)**		14.6±2.1 (15.0)	14.6±2.2 (15.0)	0.8791*
**Weight (kg)**		59.9±14.9 (57.1)	57.7±13.8 (56.6)	0.3072*
**Height (m)**		1.6±0.1 (1.6)	1.6±0.1 (1.6)	0.1824*
**Neck circumference (cm)**		32.5±3.1 (32.0)	32.4±2.9 (32.0)	0.6832*
**BMI (kg/m^2^)**		22.2±4.6 (21.3)	22.0±4.3 (21.3)	0.6716*
**Nutritional diagnosis** *** **n (%)**	**Lean**	14 (3%)	4 (2%)	0.9873**
	**Normal weight**	284 (61%)	125 (63%)	
	**Overweight**	92 (20%)	39 (20%)	
	**Obesity**	72 (16%)	32 (16%)	

Kg = kilos. m = meters. cm = centimeters.

*Student’s *t*-test of independence.

**Chi-square test.

***Classification according to Brazil [1].

In this stage of validation of the proposed formulas, considering the values of R^2^, we can conclude that the models proposed for adolescent girls and boys (Fig 1) are adequate to estimate the weight since they reached R^2^ values greater than 0.7 [30], being 0.76 for girls and 0.71 for boys (Table 6).

**Table 6:** Quality of estimates of the proposed equations for estimating adolescent weight.

	Girls (n = 125)	Boys (n = 75)
**Mean (SD) of the measured weight (kg)**	57.04 (13.30)	58.77 (14.49)
**Mean (SD) of the estimated weight (kg)**	56.47 (10.10)	58.95 (12.36)
**Difference (SD) between mean values of the measured and estimated weights (kg)**	0.57 (6.87)	-0.19 (7.03)
**99% CI difference**	(-1.04; 2.17)	(-2.33; 1.96)
**R** ^**2**^	0.76	0.71
**RMSE**	6.87	6.99
**ICC (p-value)**	0.83 (6.9×10^−34^)	0.86 (1.9×10^−24^)
**99% CI (ICC)**	(0.74; 0.89)	(0.77; 0.92)

SD = standard deviation. 99% CI = 99% confidence interval. R^2^ = coefficient of determination. RMSE = root mean square error. ICC = intraclass correlation coefficient.

Moreover, the quality of the estimates of the proposed formulas (Table 6) was checked. For both studied groups, the mean values of the measured and estimated weights are close and present a greater homogeneity in the estimated data (lower standard deviation value) in relation to the measured data, demonstrating the smoothing of the estimates. The low values of RMSE and the high values of ICC (> 0.8), confirm the accuracy of the models proposed for estimating the weight of adolescents.

To check the validation of the proposed formulas for adolescents at different stages of sexual maturity, these were tested according to such staging. Among the 125 girls in the sample used in the validation stage, 1 (1%) was at the prepubertal stage of sexual maturity (stage 1), while the others were at the pubertal stage, with 98 (78%) at stage 2 and 26 (21%) at stage 3. Among the 75 boys in the sample used for validation, 5 (7%) were at the prepubertal stage of sexual maturity (stage 1) and 70 were already at the pubertal stage, with 48 (64%) at stages 2 and 22 (29%) at stage 3. Thus, considering the low number of adolescents at the prepubertal stage, we opted for the analyses according to stages 2 and 3 of sexual maturity, pubertal stage.

The proposed equations remained adequate to estimate girls’ weight at stage 2 and boys at stage 3 (R^2^>0.7). However, in the other stage of both sexes, this adequacy was not achieved, having a 65% (R^2^ = 0.65) capacity to adjust the weight in girls at stage 3 and 68% in boys at stage 2 (Table 6), that is, less than 70% (R^2^ <0.7), which is the ideal value. However, in a positive way, the low values of RMSE and the high values of ICC (> 0.8) confirm the accuracy of the proposed equations for estimating the weight of adolescents regardless of the stage of sexual maturity. It is worth mentioning that the equations were proposed considering a global model, with a loss of accuracy and precision expected when considering only one stratum of the original population. Moreover, regardless of the stage of sexual maturity, the mean values of the measured and estimated weights are close (Table 7).

**Table 7:** Quality of the estimates of the proposed equations for estimating the weight of adolescents, according to the stage of sexual maturity.

	Girls		Boys	
	Stage 2 (n = 98)	Stage 3 (n = 26)	Stage 2 (n = 48)	Stage 3 (n = 22)
**Mean (SD) of the measured weight (kg)**	55.89 (12.62)	62.36 (14.14)	57.88 (13.50)	64.30 (14.62)
**Mean (SD) of the estimated weight (kg)**	56.0 (9.84)	59.20 (8.91)	57.64 (11.56)	65.66 (10.59)
**Difference (SD) between mean values of the measured and estimated weights (kg)**	-0.20 (6.14)	3.16 (8.73)	0.24 (7.61)	-1.36 (5.94)
**99% CI difference**	(-1.83; 1.43)	(-1.61; 7.93)	(-2.71; 3.19)	(-4.94; 2.23)
**R** ^**2**^	0.77	0.65	0.68	0.88
**RMSE**	6.11	9.12	7.53	5.96
**ICC (p-value)**	0.85 (8.1×10^−30^)	0.71 (1.4×10^−5^)	0.82 (1.8×10^−3^)	0.89 (3.2×10^−9^)
**99% CI (ICC)**	(0.76; 0.91)	(0.34; 0.89)	(0.65; 0.91)	(0.69; 0.96)

SD = standard deviation. 99% CI = 99% confidence interval. R^2^ = coefficient of determination. RMSE = root mean square error. ICC = intraclass correlation coefficient.

## Discussion

The application of multiple linear regression models used in this study indicated that the variables NC, height, and age together presented explanatory power for the variation of the measured weight above 70% in the sample of adolescent girls and above 75% in the sample of boys. These data supported the proposal of formulas for estimating weight in adolescents with a small number of variables, which are easily obtained in clinical or emergency practice. When the proposed formulas were submitted for validation, they showed high agreement, indicating that they were adequate for estimating the weight of adolescents of both sexes since they reached R^2^ values greater than 0.7 [30], being 0.76 for girls and 0.71 for boys. When validated according to the stage of sexual maturity, the formulas maintained low values of RMSE as well as high values of ICC (> 0.8), confirming their accuracy for estimating the weight of adolescents regardless of the stage of sexual maturity.

Other studies have proposed and validated formulas for estimating the weight of children and adolescents. For children, a pioneering research was carried out in 1986 in the United States by James Broselow, who developed a method for estimating weight in children based on height, called Broselow tape [20]. The study had the participation of 1,002 American children, 571 boys, and 431 girls. Lubitz et al. [20] analyzed the accuracy of this tape, and the results obtained in the linear regression analysis indicated R^2^ values of 0.94. However, when the data were grouped into weight ranges for analysis, there was a wide variation in the values of R^2^ obtained for children from 3.5 to 10 kg, 10 to 25 kg, and over 25 kg, which were 0.79, 0.86, and 0.21, respectively [20]. Broselow tape was also investigated for accuracy in a sample of 300 children aged 1 to 10 years in the city of Manchester, England. In this study, this tape had an R^2^ value of 0.85 [7]. Graves et al. [32], based on a research with Australian children, concluded that Broselow tape, although precise, can erroneously classify up to 60% of non-white children and with a higher risk in older children.

The most widespread age-based weight estimation equation is the formula developed in the United Kingdom by the Advanced Pediatric Life Support Group/European Resuscitation Council, known as the APLS formula, indicated for use in the age group from one to 10 years [33]. However, it was observed that this formula is not adequate, since it underestimates the weight in one fifth of children with an error greater than 10% [34]. In comparing the use of Broselow tape and the APLS formula to estimate weight, Argall et al. [7] reported that both methods underestimated the actual weight, with an average bias of -3.52 kg for the APLS formula and -2.74 kg for the Broselow tape.

Theron et al. [8] proposed age-based formulas to estimate the weight of children under ten years of age in New Zealand, and they reached an R^2^ of 0.78, that is, with explanatory power for the variation in measured weight of 78%.

Tinning and Acworth has also proposed Age-based formulas in order to estimate the weight of children, which are also known as Best Guess. To do this, they used data from 70,181 Australian children divided into three age groups: under 12 months, 1 to 4 years, and 5 to 14 years. Three equations were generated using regression analysis. The value of R^2^ for children under 12 months was 0.76, for the group of 1 to 4 years of age, the value was 0.60 and for the group of 5 to 14 years of age, the value was 0.65 [10].

Age-based formulas are the oldest methods for estimating weight in children. However, the weight estimated utilizing these formulas is not necessarily associated with the actual weight but with the expected weight given the principle used in the models. Thus, equations based on body measurements began to be proposed to estimate adolescents’ actual weight. In a pioneering way, Cattermole et al. [4] proposed a formula using the anthropometric variable MAC to estimate weight. The study was based on 1,370 Chinese children from 1 to 11 years old, and the proposed formula proved to be suitable for that purpose, reaching an R^2^ value of 0.96

In 2012, the Mercy Method was developed, using the humerus length and the MAC to estimate the weight [5]. To develop the Mercy Method, anthropometric data from children between two months and 16 years old, extracted from the National Health and Nutrition Examination Survey (NHANES) corresponding to 1999 to 2008, were used, totaling 17,328 children in the development stage and 1,938 in the validation. The R^2^ value obtained by this method was 0.98, higher than other previously published weight estimation methods, including the Australian Resuscitation Council equations [35] (R^2^ = 0.74), Theron et al. [8] (R^2^ = 0.7), Cattermole et al. [4] (R^2^ = 0.87), Tinning; Acworth [10] (R^2^ = 0.76), Broselow Tape (R^2^ = 0.95) [36], among others analyzed in this study.

There is also a method for estimating weight from a tape that is based on height and MAC, which has been validated in 1,800,322 children between six months and five years of age. Data were collected from nutritional surveys carried out during the years 1992 to 2017, in 51 low- and middle-income countries (not including Brazil) and with a high prevalence of acute and chronic malnutrition [19]. These authors used Kappa and Bland-Altman statistics as measures of method accuracy, and the standard deviation and the 95% confidence interval of the estimates were used as precision measures. Statistical analysis evidenced that it is an accurate and precise method.

More recently, age- and MAC-based weight estimation formulas have been proposed from a sample of 861 Argentinian children aged between 2 and 18 years. These formulas achieved an R^2^ value of 0.87 for females and 0.92 for males, and the Bland-Altman plots showed strong consistency between observed and predicted weight values [21].

In this study, the proposed and validated formulas are for estimating the weight of adolescents, including the age group of 10 to 19 years. It is noted that there are no predictive equations including this age group that use NC, making such formulas attractive as they fill a gap of need in clinical and emergency practice.

There are indeed other methods available and suitable for estimating weight;however, the formulas proposed here, in addition to covering an age range not covered by other studies, were created based on a national database, which makes the sample more accurate as to the economic, social and ethnic characteristics of Brazilian adolescents. Proposing and validating new equations based on body measurements capable of predicting current weight and based on a representative sample of Brazilian adolescents greatly benefit national clinical practice.

## Study limitations

The formulas proposed here were validated in a sample of healthy adolescents and may present limitations of precision and accuracy when applied to adolescents with body deformities. The present equations were originated on Brazilian population. Caution should be exercised when applying these equations to adolescents of other countries. The equations were based on data from previously sampled population that possibly vary widely. Because of this, caution should be exercised when it is used at an individual clinical level.

## Strengths

It is one of the largest cross-sectional studies to develop weight equations for adolescents, to our knowledge, the first to design equations to estimate weight exclusively in adolescents and developed for low- and middle-income counties where it is more difficult to access appropriate scales in emergency care settings. Weight, can be estimated in adolescents when it, cannot be obtained directly. Another advantage is that the equations designed to estimate weight uses NC, which is a simple and reliable measure. Furthermore, the equation includes adolescents from 2 to 19 years of age, making it useful for the full pediatric age range.

## Conclusion

The proposed formulas use a small number of easily obtainable variables, which indicates the practicality and feasibility of using them safely. They present adequate accuracy and precision, and for these reasons they are a viable option for estimating the weight of Brazilian adolescents unable to have their weight measured.

Complementary studies are required to assess the applicability of these formulas for estimating weight in adolescents from Brazil and other countries.
